# The Application of Single-Cell Technologies in Cardiovascular Research

**DOI:** 10.3389/fcell.2021.751371

**Published:** 2021-10-11

**Authors:** Yinan Chen, Yang Liu, Xiang Gao

**Affiliations:** ^1^Fuwai Hospital, Chinese Academy of Medical Sciences, Shenzhen, China; ^2^State Key Laboratory of Cardiovascular Disease, Fuwai Hospital, National Center for Cardiovascular Diseases, Chinese Academy of Medical Sciences and Peking Union Medical College, Beijing, China; ^3^Department of Vascular Surgery, The Second Hospital of Hebei Medical University, Shijiazhuang, China

**Keywords:** single-cell omics, cardiovascular research, cardiovascular disease, single-cell sequencing, epigenetics, genetics

## Abstract

Cardiovascular diseases (CVDs) are the leading cause of deaths in the world. The intricacies of the cellular composition and tissue microenvironment in heart and vasculature complicate the dissection of molecular mechanisms of CVDs. Over the past decade, the rapid development of single-cell omics technologies generated vast quantities of information at various biological levels, which have shed light on the cellular and molecular dynamics in cardiovascular development, homeostasis and diseases. Here, we summarize the latest single-cell omics techniques, and show how they have facilitated our understanding of cardiovascular biology. We also briefly discuss the clinical value and future outlook of single-cell applications in the field.

## Introduction

The transcriptome and epigenome are important determinants of the proteome, the latter of which confers functional specificity to individual cells within the tissue. As cellular composition dictates tissue function, understanding cellular heterogeneity is critical to deciphering organ homeostasis and disease progression. Traditional sequencing methods, such as bulk RNA-seq and bulk ATAC-seq, provide only an average readout of pooled cell populations, which masks cellular heterogeneity, and is incapable of identifying specific cell types.

In the past decade, various powerful single-cell techniques have been developed, enabling scientists to interrogate single cells at multiple molecular levels. At present, there are single-cell RNA-sequencing (scRNA-seq) and single-nucleus sequencing (snRNA-seq) to study gene expression (Shapiro et al., 2013; Islam et al., 2014; Hu P. et al., 2018), single-cell assay for transposase-accessible chromatin sequencing (scATAC-seq) to study DNA accessibility (Buenrostro et al., 2015b) and single-cell DNA methylome sequencing to investigate DNA methylation at single cell resolution (Luo et al., 2017). Building upon these techniques, single-cell multi-omics, which is a combination of at least two of the above techniques applied to a single cell, provides unprecedented resolution to investigate the interconnectedness of molecular regulatory mechanisms, and promises more accurate identification of cell subpopulations and cell states (Guo et al., 2017; Hu Y. et al., 2018).

Cardiovascular diseases are the leading cause of death globally. Deeper understanding of the cellular makeup and molecular processes in the heart and the vasculature is necessary for dissecting disease mechanisms and improving of therapeutic strategies. Single-cell techniques are tremendously useful for uncovering cellular diversity, revealing cell–cell interactions, identifying potential biomarkers, and delineating disease dynamics (Li et al., 2017; Macaulay et al., 2017; Davie et al., 2018; Jackson et al., 2020), particularly when different single-cell omics data are integrated. In this review, we discuss the current single-cell technologies and platforms, including their advantages and weaknesses. We also summarize recently published single-cell studies in the cardiovascular field.

## Single-Cell Technologies

### Single-Cell RNA Sequencing

Single-cell RNA sequencing (scRNA-seq) is by far the most widely used single-cell technology in cardiovascular biology. It is a powerful tool for analysis of all transcripts in a single cell. The typical workflow of a scRNA-seq experiment includes single-cell capture, reverse transcription, amplification, library preparation, sequencing and data analysis (Potter, 2018). Since the publication of the first single-cell transcriptomics study in 2009 (Tang et al., 2009), a variety of scRNA-seq techniques, have been developed (Svensson et al., 2018). Each variation of scRNA-seq has its own advantages and drawbacks, thus, choosing the appropriate method is highly dependent on tissue/cell type and experimental design (Table 1). For example, single cell selection approaches such as micropipetting and laser capture microdissection allow the visualization of cells prior to selection, but are laborious and low-throughput, and are prone to bias. By contrast, droplet-based cell selection techniques are high-throughput, suffer from limitations including cell size restraint [not suitable for adult cardiomyocytes (CMs)] and “blind” selection of cells. The latter attribute therefore requires high input cell quantity (10^5^–10^6^ cells per sample) and quality (>80% viability) (Gao et al., 2020). One way to circumventing cell size restraints is the use of nuclei instead of intact cells. Single-nucleus RNA sequencing (snRNA-seq) thus reduces the technical challenge of isolating adult CMs, and minimizes stress-induced aberrant gene expression (Hu P. et al., 2018; Cui et al., 2020). It is worthwhile to note, however, while most studies found comparable clustering of cell populations with scRNA-seq and snRNA-seq (Bakken et al., 2018; Slyper et al., 2020), adult CMs are different from most other cell types in that they can contain more than one nuclei, which may confound data interpretation (Ding et al., 2020). Microwell-based selection approaches (e.g., ICELL8) come with the advantage of visualizing cells and staining them for viability before capture. In addition, the large nozzle diameter does not limit target cell size (i.e., compatible with adult CMs). However, these methods are considered medium-throughput, capturing approximately 2,000 cells per chip.

**TABLE 1:** Main features of the single-cell technologies.

**Methods**	**Target**	**Features**	**References**
		- Full-length transcript sequencing	
Smart-seq2	Transcriptome	- Detect more transcripts than 10x Genomics - Low throughput	Picelli et al., 2013

		- Full-length transcript sequencing	
Smart-seq3	Transcriptome	- Use 5′ unique molecu_x0002_lar identifier (UMI) - Higher sensitivity than Smart-seq2 - Low throughput	Hagemann-Jensen et al., 2020

		- High throughput: test 8 samples simultaneously, capture 500-10000 cells for each sample	
		- Low cost	
		- One-day workflow	
lOx Genomics	Transcriptome	- “Blind” selection of cells - Require high input cell quantity (10^5^–10^6^ cells per sample) and quality (> 80% viability) - Require cell size < 30 μm - Serious dropout problem	Gao et al., 2020

CEL-seq2	Transcriptome	- Perform with Fluidigm Cl microfluidics system - 3′-end sequencing	Hashimshony et al., 2016

		- Perform either in 96/384-well plates or with Fluidigm Cl micro fluidics system	
		- 5 μm < cell size < 25 μm	
STRT-seq	Transcriptome	- 5′-end sequencing - 2 to 3-day workflow - Require maximum 5000 cells for one test	Natarajan, 2019

		- Use Smart-seq2 system	
		- Perform both 3′-end sequencing and full-length transcript sequencing	
		- Test 8 samples simultaneously	
ICELL8	Transcriptome	- Throughput lower than 10x Genomics, capture ∼2000 cells for each chip - Require high input cell quality (>70% viability) - Require ∼20000 cells for each test - Visualization of cell selection	Goldstein et al., 2017

MDA	Genome	-Require 1000 pg DNA - 70% coverage rate of genome - Does not support CNV analysis	Dean et al., 2002

MALBAC	Genome	- Require 0.5 pg DNA - ∼90% coverage rate of genome - Low bias - Support CNV analysis	Zong et al., 2012

sci-ATAC-seq	chromatin accessibility	- Support 1500 cells for sequencing - With median reads of 2,500 per cell - Collision rate: ∼11%	Cusanovich et al., 2015

IFC scATAC-seq	Chromatin accessibility	- Only perform 96 cells simultaneously - 70,000 reads per cell	Buenrostro et al., 2015b

lOx Genomics	Chromatin accessibility	- High throughput - Low cost - Compatible with fresh, fixed (methanol), and cryopreserved single cell or singlenuclei suspensions - Simple workflow	Satpathy et al., 2019

scRRBS	DNA methylation	- High sensitivity - Epigenetic marker detected: 5mC - Cover 1.5 million CpG sites per cell - RRBS strategy - Focus on the enrichment of CpG-rich regions	Guo et al., 2015

scBS-seq	DNA methylation	- Low DNA input (˜100 ng) - Epigenetic marker detected: 5mC - Cover 3.7 million CpG sites per cell - PBAT strategy	Miura et al., 2012

		- Spatial information not available	
CyTOF	Proteome	- 42 metal isotopes available - Cell size < 30 μm - Require 1-3 million cells for each sample	Cheung and Utz, 2011

		- Spatial information available	
		- 35 metal isotopes available	
IMC	Proteome	- Support FFPE and cryopreserved tissues - Use laser system to ablate tissue, 1 μm image resolution - Re-analysis not available	Giesen et al., 2014

		- Spatial information available	
		- Detect 40+ proteins simultaneously	
MIBI	Proteome	- Support FFPE and cryopreserved tissues - Based on secondary ionization mass spectrometry, 250 nm image resolution - Supprot re-analysis	Keren et al., 2018

		- ELISA-based approach	
SCBC	Proteome	- Spatial information not available - Require only a few hundred cells	Shi et al., 2012

		- Excellent tolerance for salts	
MALDI	Metabolome	- High sensitivity and throughput - Combined with flash-freezing sample preparation method, can reflect the natural distributionof metabolites	Dueñas et al., 2017

		- Use optical microscope, visualization for cell selection	
LSCMS	Metabolome	- Detect 100-1000 of molecular peaks from a single living cell - Combined with a motorized x-y stage to an automated software system, can select and pick up desired cells automatically	Shimizu et al., 2015

Different sequencing protocols utilize different strategies to capture, amplify and sequence mRNA molecules. Some protocols generate a strong bias toward either the 5′-end [e.g., Single-cell tagged reverse transcription (STRT-seq)] (Natarajan, 2019) or the 3′-end (e.g., Drop-seq), cell expression by linear amplification and sequencing 2 (CEL-seq2) (Hashimshony et al., 2012; Bageritz and Raddi, 2019; Natarajan, 2019). These are commonly used for their high-throughput and relatively low costs. However, if the goal of the experiment goes beyond quantification of gene expression, then one may use full-length transcript sequencing [e.g., Switching mechanism at 5′ end of the RNA transcript sequencing (Smart-seq2) (Picelli et al., 2013), Smart-seq3 (Hagemann-Jensen et al., 2020)] to obtain a greater coverage of transcripts. Moreover, full-length sequencing allows characterization of alternative splicing, single-nucleotide variants, transcription start sites, and the detection of monoallelic and imprinted genes (Ramsköld et al., 2012; Picelli et al., 2014; Volden et al., 2018; Picelli, 2019).

### Other Single-Cell Omics

In addition to the transcriptome, other ensembles of molecules (e.g., genome, proteome, and metabolome) and cellular or molecular states (e.g., chromatin accessibility and methylome) of can also be profiled at single-cell level. Techniques such as single-cell chromatin accessibility sequencing (scATAC-seq), proteomics and DNA methylomics are now widely used either alone, or to complement transcriptomic data (Table 1) (Tanay and Regev, 2017; Efremova and Teichmann, 2020).

The aim of genomics is to study an organism’s complete set of DNA (genome), including its structure, function, evolution, and the impact of its changes on organisms. Single-cell whole-genome sequencing is generally accomplished via the two major whole genome amplification (WGA) methods, including multiple displacement amplification (MDA) (Dean et al., 2002), multiple annealing and looping based amplification cycles (MALBAC) (Zong et al., 2012). As one of the pioneering WGA techniques, PCR-based methods [e.g., degenerate oligonucleotide-primed polymerase chain reaction (DOP-PCR)] are less susceptible to DNA sample quality, but suffer from several limitations, which include inadequate average product size, non-specific amplification biases and incomplete genome coverage. MDA is an isothermal technique that utilizes the ϕ29 DNA polymerase for its exceptional fidelity (due to its 3′→5′ proofreading exonuclease activity), strand-displacement activity and processivity (up to 70–100 kb without dissociation from template). Thus, MDA reduces the amplification bias from 40-fold to less than 3-fold and achieves much gearter genome coverage (∼70%) (Dean et al., 2002). The more advanced MALBAC relies on quasilinear DNA preamplification, which is used to reduce bias associated with non-linear amplification, followed by exponential amplification (regular PCR) to acquire sufficient material for sequencing. This design yields a more even representation of the genome (i.e., uniformity) and better reproducibility. However, both of these two techniques have their own limitations. For MDA, the irreproducible sequence-dependent bias and production of chimeric products are the protential limitations; for MALBAC, it is the high false positive rate for single-nucleotide variations (SNVs) detection (Huang et al., 2015).

ATAC-seq uses hyperactive Tn5 transposase to tag and fragment DNA sequences in open chromatin regions simultaneously, thereby identifying chromatin accessibility (Buenrostro et al., 2015a). Initially, three major strategies for scATAC-seq were developed: combinatorial cellular indexing (e.g., sci-ATAC-seq), microfluidics-based approach using an integrated fluidics circuit (IFC), and droplet-based approach (Buenrostro et al., 2015b; Cusanovich et al., 2015; Satpathy et al., 2019). In sci-ATAC-seq, the nuclei of lysed cells are labeled by unique combinations of two barcodes during transposition and PCR amplification, respectively. This approach allows sequencing of approximately 1,500 cells with median reads of 2,500 and at a collision rate of ∼11%. IFC scATAC-seq enable get more than 70,000 reads per cell (Buenrostro et al., 2015b). In droplet-based ATAC-seq, single nuclei are prepared using the 10X Genomics protocol, transposed, and are loaded onto a microfluidic chip for generating barcoded GEMs (gel bead in emulsion). The throughput of each sample well on a microfluidic chip is up to 10,000 single nuclei. The capture ratio of this method is more than 65%. Building upon these three strategies, other scATAC-seq methods have emerged, such as Perturb-ATAC (Rubin et al., 2019) and plate-based scATAC-seq (Chen et al., 2018). Since this method provides a genome-wide landscape of chromatin accessibility at single-cell resolution, scientists are able to identify cell types, define genomic features, such as *cis*-regulatory elements (e.g., promoters and enhancers) and trans-regulatory elements (e.g., transcription factors) and build gene regulatory networks with it. Gene activity and accessibility to genetic variants can also be derived using scATAC-seq data (Satpathy et al., 2019). Furthermore, integration of single-cell transcriptome and chromatin accessibility data may improve cell identity annotation (Jia et al., 2018). Specifically, joint analysis of these two types of omics data facilitates the detection of correlations between *trans*- and *cis*-regulatory elements and the cellular state of interest (Stuart et al., 2019). However, the noisy and sparse nature of scATAC-seq signals remains a big challenge for computational analysis.

DNA methylation is an important epigenetic modification that establishes patterns of gene repression. Currently, there are two approaches for single-cell DNA-methylome profiling. The first method is single-cell reduced-representation bisulfite sequencing (scRRBS) (Guo et al., 2015). This protocol integrates the steps from *Msp*I digestion to bisulfite conversion into one tube of cell lysate, thus minimizing DNA loss and gathering methylation information on approximately 1 million CpG sites within an individual cell. The other method, single-cell bisulfite sequencing (scBS-seq), employs a modified post-bisulfite adapter tagging (PBAT) approach (Miura et al., 2012; Smallwood et al., 2014). This approach allows for a lower starting amount of DNA (∼100 ng) (Miura et al., 2012). Compared to scRRBS, scBS-seq is capable of measuring DNA methylation at up to 48.4% of the CpG sites and achieves higher recovery rates simultaneously (Smallwood et al., 2014; Schwartzman and Tanay, 2015).

Proteomics has been used for decades to study the characteristics of all proteins in a sample in large-scale, including protein expression level, posttranslational modification and protein–protein interaction (Aslam et al., 2017). However, it is not until recent years that single-cell protein and proteomic techniques were developed, offering opportunities to analyze the functional states of individual cells. Mass cytometry, also known as CyTOF (cytometry by time-of flight) permits cytometric measurement of up to 42 proteins per cell by using time-of-flight mass spectrometry and heavy-metal tagged antibodies, and theoretically can detect up to 100 isotopes (Cheung and Utz, 2011). It is a potentially powerful tool for immune-monitoring and clinical diagnosis. Many studies have demonstrated its applicability in clinical prognosis and diagnosis (Amir et al., 2013; Behbehani et al., 2015; Ferrell et al., 2016; Fisher et al., 2017). Imaging mass cytometry (IMC) and multiplexed ion beam imaging (MIBI) are two techniques related to CyTOF. IMC is in essence a combination of immunohisto(cyto)chemistry and mass cytometry, made possible through the integration of a laser ablation device (Giesen et al., 2014). Stained tissue sections and cells are almost ablated by a pulsed laser pixel by pixel, where associated metal isotopes are measured and indexed. Currently, IMC offers 1 μm-image resolution and requires almost 50 copies of an epitope per pixel for minimal detection. Different from laser-based IMC, MIBI is based on secondary ionization mass spectrometry, only a few hundred nanometers of tissue are lost during a MIBI scan, which means that the stained tissue can be re-analyzed multiple times with this technique (Keren et al., 2018). When combined with TOF detection system, MIBI-TOF can measure a large scale of atomic masses (Keren et al., 2018, 2019). This instrument has an image resolution of 250 nm and a rate reaching 10,000 pixels per second. The major advantage of IMC and MIBI is that they can monitor protein modifications, such as histone acetylation and kinase phosphorylation, with spatial information. Single-cell barcode chip (SCBC) is an ELISA-based approach, it allows detection of more than 40 proteins per cell and provides data with deep depths (Shi et al., 2012). Only a few hundred cells are required for each assay. The advantage of this technique is that single cells can be cultured in the microchambers on the chip, and is therefore suitable for the study of secreted protein and paracrine (Xue et al., 2015).

The term metabolomics was first proposed in 2001, and is defined as the comprehensive and quantitative analysis of all metabolites (small molecules, typically less than 1 kDa, including nucleosides, lipids, amino acids and carbohydrates) of the biological system (Fiehn, 2001). The metabolite heterogeneity of individual cells from tissue or organ reflects stochastic biochemical processes, cell cycle stages, environmental stress, and diseased states. However, analyzing the metabolomes of single cells is technically extremely challenging owing to the chemical diversity and instability of metabolites, as well as the low amount input material because metabolites cannot be amplified. Thus, both the resolution and the sensitivity of the analytical method are important points to consider. At present, mass spectrometry (MS)-based techniques are by far the most popular methods to analyze single-cell metabolomes. Typically, the contents of isolated single cells are processed via a separation- or non-separation-based approach, before subjecting them to MS analysis. Currently, a variety of ionization techniques have been applied to single-cell metabolomics, including time-of-flight secondary ion mass spectrometry (TOF-SIMS) (Kleinfeld et al., 2004), matrix-assisted laser desorption-ionization (MALDI) (Dueñas et al., 2017), nanostructure-initiator MS imaging (NIMS) (O’Brien et al., 2013), live single-cell mass spectrometry (LSCMS) (Shimizu et al., 2015), and laser-ablation electrospray ionization mass spectrometry (LAESI-MS) (Taylor et al., 2021). Among them, MALDI and LSCMS are the most frequently used methods. In MALDI, cells are co-crystallized with matrix (a solution that supplies protons) on a metal plate and are irradiated and ionized by a UV laser beam. The ions are then analyzed depending on mass-to-charge rations (*m/z*). The sensitivity of this method is good enough to be used in single-cell analytical studies. However, the crystalized condition of cells and the vacuum environment on the metal plate is not sufficiently physiologically relevant, hence, doubts were raised whether MALDI causes metabolite distortion or exhaustion of molecules. In addition, lipids dominate MALDI ionization, which consequently impacts the detection of other metabolites. Compared with MALDI, LSCMS enables direct and real-time analysis of molecules at single-cell resolution. Living cells are monitored on a video microscope, allowing behavior- and morphology-based cell selection. Cells are kept in a suitable medium until ionization, a protocol that maximally preserves the natural cellular environment.

Despite the rapid development of single-cell omics, the studies depend on a certain single omics technique are unable to draw a complete picture of the regulatory networks in a cell. Therefore, single-cell multi-omics techniques, which are combinations of simultaneous measurements of the genome, transcriptome, proteome, or epigenome from individual cells, has gained momentum for its capability to directly study the correlations between genetic and phenotypic changes. At present, single-cell multi-omics technologies can be generally divided into four classes: (1) transcriptome and genome, for investigating the relationship between gene expression and genomic alterations, includes DR-seq (Huo et al., 2016), G&T-seq (Macaulay et al., 2015), SIDR (Han et al., 2018), TARGET-seq (Chaligne et al., 2019), and scTrio-seq (Hou et al., 2016); (2) transcriptome and DNA methylation, for exploring the relationship between gene expression and DNA methylation, includes scM&T-seq (Angermueller et al., 2016), scMT-seq (Hu et al., 2016), scTrio-seq (Hou et al., 2016), and scNMT-seq (Clark et al., 2018); (3) transcriptome and chromatin accessibility, for studying relationship between gene expression and chromatin accessibility, includes sci-CAR (Cao et al., 2018), SNARE-seq (Chen et al., 2019), scNMT-seq (Clark et al., 2018); (4) transcriptome and proteome, for discovering the relationship between gene expression and protein expression, includes PEA/STA (Genshaft et al., 2016), PLAYR (Frei et al., 2016), CITE-seq (Stoeckius et al., 2017), REAP-seq (Peterson et al., 2017), RAID (Gerlach et al., 2019), and ECCITE-seq (Mimitou et al., 2019).

## Application of Single-Cell Technologies in Characterizing Cellular Heterogeneity in the Heart

### Cellular Heterogeneity of the Developing Heart

On a temporal axis, the heart undergoes significant changes in cellular composition and function during development and disease progression (Harris and Black, 2010; Espinoza-Lewis and Wang, 2012; Buijtendijk et al., 2020). During development, the cardiac cellular composition undergoes drastic changes as multipotent cells make multiple step-wise decisions to differentiate into various states (DeLaughter et al., 2016; Li et al., 2016; Scialdone et al., 2016). Homeobox genes *Nkx2.5* and *Isl1*, expressed by cardiac progenitor cells (CPCs), play critical roles in heart formation and development during early embryonic stages (Moretti et al., 2006; Wu et al., 2006). By integrating scRNA-seq and scATAC-seq to map the developmental trajectories of Nkx2.5+ and Isl1 + CPCs in early (E7.5, E8.5, and E9.5) mouse embryonic hearts (Jia et al., 2018), Jia et al. (2018) revealed that Isl1 + CPCs pass through an attractor state before separating into different developmental branches, while extended expression of *Nkx2.5* commits CPCs into a unidirectional cardiomyocyte (CM) fate. Interestingly, cells within the same cluster defined by transcriptomic sequencing possessed different chromatin accessibility. CPC fate transitions were associated with distinct chromatin states, which is critically dependent on Isl1 and Nkx2.5 (Jia et al., 2018). Notably, scATAC-seq exhibited higher sensitivity toward delineating cellular heterogeneity than scRNA-seq, because it detected five subpopulations of Isl1 + CPCs at E8.5 and E9.5, while the latter only detected three (Jia et al., 2018). Therefore, integration of different types of data may improve the resolution with which we define cellular states and events.

Recently, Cui et al. (2019) systematically revealed the transcriptional landscape of human fetal heart during development at single-cell resolution. They identified four main cell types in human embryonic heart, including CMs, fibroblast-like cells, endothelial cells and valvar cell, as well as some other cell types, such as smooth muscle cells and immune cells (e.g., macrophages, T cell, B cells). During development, the proportion of CMs in the atrium and ventricle dramatically declined, whereas the proportion of NCMs, such as fibroblast-like cells and macrophages, increased gradually, accompanied by a set of up-regulated ECM related genes, including *DCN*, *COL1A1*, and *LUM*, indicating a critical role of NCMs in heart development. In addition, in the developing heart, CMs experiences a transition from loose trabecular CMs to a more mature and compact CMs. This study identified the gene expression changes between these two states. The authors compared the transcriptional changes in different cardiac chambers, and discovered that the difference arose as early as 5 weeks. New knowledge provided by this work deepens our understanding of human heart development and may provide us some inspiration on the differentiation of mature functional cardiac cells *in vitro* from stem cells.

An even more precise spatiotemporal map of the developing human heart was accomplished by the integration of single-cell transcriptomics, spatial transcriptomics (ST) and *in situ* sequencing (ISS) of four human developmental hearts across three time points (Asp et al., 2019). At first, with ST, the authors investigated unique gene expression patterns in each anatomical region, classified cells of human developing heart into 10 clusters and depicted global spatiotemporal information at the three time points. Then, through scRNA-seq, the authors characterized cellular heterogeneity of human fetal heart and classified cells into 15 clusters, which was incorporated the result from ISS. The result of ISS was not only consistent with ST and scRNA-seq data, but also complemented other methods. For example, analysis of the spatial position and functional heterogeneity of epicardium and epicardium-derived cells (EPDCs), four fibroblasts clusters and two endothelial cells clusters, could not be accomplished by scRNA-seq. Finally, the sections of ST and the sections of ISS were aligned to construct two types of 3D models of human fetal hearts. Compared with cellular spatial distribution predicted via ST, ISS provided spatial information at a finer structural resolution. This work exemplifies how incorporation of various RNA sequencing approaches can facilitate our comprehension of heart development in different dimensions, a strategy that could be extended to other organs to decipher the global process of human development.

### Cellular Heterogeneity of the Adult Heart

Hu P. et al. (2018) investigated the transcriptional landscape of postnatal maturing mouse hearts in both healthy states and in a pediatric mitochondrial cardiomyopathy model by snRNA-seq. Based on gene expression signatures, they classified CMs into developing and mature CMs, each of which encompass several subpopulations. Gata4 and Myocd are critical transcription factors for CM development (Huang et al., 2012; Borok et al., 2016). The authors found that *Gata4* and *Myocd* were highly expressed in developing CMs, but not in mature CMs or non-myocyte cells, indicating that the function of these two transcription factors were confined to a specific CM population. In addition, they discovered a small population of presumably proliferating CMs, which express cell cycle genes, including *Mki67*, *Cenp*, and *Kif15*. Compared with P6 control mice, the percentage of this population in P10 declined from 5.5 to 1.8%, whereas the percentages of other cell types remained relatively stable. Mitochondrial cardiomyopathy is defined as cardiomyopathy caused by mitochondrial DNA mutations (Ozawa, 1994). In this study, dramatic cell type-specific and subtype-specific transcriptional remodeling occurred in mice with mitochondrial cardiomyopathy. This study provided some of the first insights into the postnatal developing heart at single-cell resolution, and shed light on cell type alterations in mitochondrial cardiomyopathy. Wang L. et al. (2020) investigated, for the first time, the heterogeneity of adult human heart and compared the distinct cellular compositions among healthy hearts, hearts with heart failure (HF), as well as hearts partially recovered from HF, at single-cell resolution (Wang L. et al., 2020). This is the first and only high-throughput sequencing study of intact adult human CMs. The authors revealed distinct cellular compositions and functions in different anatomic regions of the heart. For example, atrial CMs possess secretary capability, whereas the CMs in left ventricle (LV) were mainly responsible for lipid metabolism. Furthermore, different LV CM clusters express distinct sets of genes for contraction and metabolism, indicating the functional heterogeneity of CMs in the LV.

Recently, Litviňuková et al. (2020) characterized the molecular signatures of six anatomical regions of the heart (left and right ventricular free walls, left and right atrium, the left ventricular apex, and interventricular septum) from 14 adult hearts, using snRNA-seq for CMs and scRNA-seq for the rest of the cell types. Cells were classified into 11 major populations comprising atrial cardiomyocytes, ventricular cardiomyocytes, fibroblasts and smooth muscle cells. When studying cardiomyocytes specifically, the authors found that the percentage of ventricular cardiomyocytes was higher in female versus male hearts, and that the percentage of cardiomyocytes in ventricles was higher than that in the atria. Furthermore, ventricular cardiomyocytes expressed more sarcomeric proteins, including MYH7 and MYL2, than their atrial counterparts. The latter was enriched in the expression genes such as *HAMP*, *ADLH1A2*, and *ROR2*, indicating significant roles of iron homeostasis (Huang et al., 2020), retinoic acid synthesis and Wnt signaling pathway (Mazzotta et al., 2016) in atrial function. These findings shed light on chamber-specific cardiomyocyte molecular signatures related to sex differences and cardiac function.

## Application of Single-Cell Technologies in Studying Cardiac Cell–Cell Interactions

Cell–cell communication has been shown to be central to many biological processes, including embryonic development and diseases. Ligand-receptor pairs, as a common way of cell–cell communication, have been found to play crucial roles in the heart. For instance, endothelial EphB4 and its ligand ephrin-B2 are important regulators of vascular morphogenesis and arteriovenous differentiation during development (Pitulescu and Adams, 2010); activin type II receptor and its ligands (e.g., activin A, GDF8 and GDF11) have been shown to participate in regulating cardiomyocyte function and HF progression (Roh et al., 2019). However, conventional experiments are limited in scale and throughput, examining only a targeted set of upstream and downstream molecules of a certain pathway. By contrast, with single-cell techniques, we can construct cell-cell interaction networks, discover putative ligand-receptor pairs and signaling pathways. When characterizing the adult human heart in healthy individuals and HF patients, Wang L. et al. (2020) discovered that non-cardiomyocytes (NCMs) played a central role in influencing cardiomyocyte biology and shaping cardiac function through ligand-receptor interactions. In particular, a fibroblast subpopulation enriched for functions related to extracellular matrix organization displayed the highest frequency of putative interactions with other cell types in the left atrium (LA), whereas an endothelial cell (EC) subcluster (ACKR1+) involved in cytokine production and chemokine secretion, showed the greatest influence in the left ventricle (LV). While most ligand-receptor pairs did not distinguish between chambers, some were specific, and were indicative of the distinct functions of the atrium and the ventricle. Furthermore, NCMs, especially the ACKR1 + -EC population was implicated in regulating cardiac function. The most abundant ligands secreted by this EC cluster were associated with maintenance of heart contraction. Importantly, injection of ACKR1 + ECs into the infarcted region of mouse hearts significantly retarded the decline in cardiac function. These observations highlight the regulatory role of NCMs in cardiac homeostasis, and their translational value.

Likewise, single-cell gene expression profiling of NCMs in adult mouse heart revealed a dense network of intercellular communication that is critical for heart homeostasis (Skelly et al., 2018). Fibroblasts (FBs) were identified as the most trophic cell population with dense connections to other cell types. For example, factors expressed and secreted by FBs, including Csf1 (Braza et al., 2018) and Vegfa (Han et al., 2019), indicate that they support both cardiac macrophage and EC growth. Furthermore, this study also revealed multiple cell populations participating in the nervous innervation of the heart. Ngf and Ntf3 are both key factors for axonal development (Usui et al., 2012; Crerar et al., 2019; Li et al., 2020). Their expression by cardiac pericytes and fibroblasts suggests the potential role of pericytes and fibroblasts in the development of the autonomic nervous system in heart. Investigating cardiac cell crosstalk networks at single-cell resolution underscores the contributions of different non-myocyte cell types and subtypes in cardiac homeostasis, and suggests potential nodes of regulation that could be exploited for therapeutic purposes.

## Application of Single-Cell Technologies in Heart Disease

At present, single-cell technologies have been widely used in heart disease studies, especially single-cell RNA-seq and single-cell ATAC-seq. Recently, Alexanian et al. (2021) utilized scRNA-seq and scATAC-seq to analyze bromodomain and extra-terminal domain (BET) inhibitor JQ1- and vehicle-treated mouse hearts that underwent transverse aortic constriction (TAC). They discovered that the inhibitor of BET, through inhibiting the transition of fibroblasts into myofibroblast, attenuates heart fibrosis. They showed that the transition of fibroblasts was activated by transcription factor *Meox1* and that the enhancer of *Meox1* was regulated by BET. These findings suggest that *Meox1* may be a new therapeutic target for HF and cardiac fibrosis.

Recently, using scRNA-seq and scATAC-seq, Wang et al. compared the gene expression and chromatin accessibility in regenerative and non-regenerative hearts with or without ligation of left anterior descending (LAD) artery (Wang Z. et al., 2020). They uncovered gene regulatory networks responsible for the regenerative responses to injury in the neonatal heart. They found that: (1) after myocardial infarction (MI), the accessibility of *cis-*regulatory elements was significantly different between regenerative and non-regenerative hearts, especially in fibroblasts, the most injury-sensitive cell type; (2) the epicardium contributed in the regenerative response to injury, indicating its special function in the neonatal heart; (3) the epicardium may stimulate angiogenesis during neonatal heart regeneration through binding of its specialized ligand RSPO1 with endothelial cell (EC) receptors LRP6 and LGR4, consequently activating the Wnt/beta-catenin signaling pathway; (4) the proportion of macrophages and monocytes was elevated after injury. They secrete cardiotrophin like cytokine factor 1 (CLCF1) that participates in the regenerative process. This study provides a comprehensive transcriptomic and epigenomic database for neonatal mouse heart regeneration after injury, and offers clues for therapeutic targets of cardiac injury.

Aside from adult cardiac diseases, scRNA-seq has also been applied to study congenital disorders. In investigating the cellular basis for cardiac malformation, de Soysa utilized scRNA-seq coupled with a Boolean network-based lineage-specifier prediction method, and predicted *Irx4* and *Plag1* as specifiers of right ventricle (RV) cells, while *Hand2*, *Tead2*, and *Arid3b* were crucial for outflow tract (OFT) cell-fate determination. Transcriptional dysregulation caused by loss of *Hand2* preceded any morphologic defect. In the absence of *Hand2*, OFT-fated cells were unable to specify, whereas properly specified RV-fated-cells failed to differentiate, leading to severe cardiac defects. This work demonstrates the power and suitability of single-cell techniques in revealing the molecular basis in early cardiogenesis.

## Application of Single-Cell Technologies in Vascular Diseases

### Atherosclerosis

Atherosclerosis (AS) is considered an inflammatory disease involving complex crosstalk between immune and vascular cells (Libby, 2012; Tabas and Lichtman, 2017; Tay et al., 2019). Prior to the application of single-cell techniques, many studies have tried to demonstrate the complex composition and function of leukocytes in atherosclerosis. Macrophages were considered the most abundant leukocytes in any type of lesion and the most significant factor for the size of lesion (Hansson and Libby, 2006). Among the many types of macrophages, pro-inflammatory M1 macrophages are mainly non-foamy cells and express inflammatory markers, such as TNF, NLRP3, ZPF36, IL1β, CXCL2, and CCL2, and are therefore considered as the main mediator of inflammation in lesions (Willemsen and de Winther, 2020). Anti-inflammatory M2 macrophages can be further divided into M2a, M2b, and M2c subtypes (Colin et al., 2014). TREM2^hi^ macrophages, characterized by excessive uptake of lipids, seems to exhibit the M2 phenotype (Willemsen and de Winther, 2020). Various types of T cells were detected in atherosclerotic plaques. For example, T helper 1 (T_H_1) cells play pro-atherogenic roles, whereas regulatory T (T_reg_) cells play anti-atherogenic roles in plaques. However, T_reg_ cells can alter their phenotype and turn pro-atherogenic (Sawant and Vignali, 2014). Yet the roles of other T cells, such as T_H_2, T_H_9, T_H_17, T_H_22, follicular helper T cells, and CD8^+^ T cells in atherosclerosis are still unclear. Hence, accumulating studies are unraveling the cellular diversity in atherosclerosis, single-cell omics are still necessary to obtain unbiased profile cell types in AS and predict their functional implications. With CyTOF, CITE-seq, and scRNA-seq, Fernandez et al. (2019) characterized immune cells in carotid artery plaques and blood samples from symptomatic (SYM) or asymptomatic (ASYM) AS patients. They discovered that: (1) in plaques, the quantity of CD8^+^ T cells were much higher than CD4^+^ T cells; (2) compared with blood, some T cells clusters (MC11, MC12, and MC20) in plaques expressed more PD-1 (programmed cell death protein 1, the marker of T cell exhaustion), indicating that T cell exhaustion may be caused by the inflammatory microenvironment; (3) genes expressed in plaque T cells were associated with inflammation, differentiation, and cell proliferation, whereas the gene expressed by T cells in the blood were related to inhibition of T cell function; (4) dysfunction of T cells and macrophages in plaques was a crucial driver for cardiovascular (CV) events. This work shed light on critical role of immune cells in clinical CV events therapy and hinted toward new targets for AS treatment.

In addition, combined with transgenic mice, scRNA-seq enables to further illustrate the role of certain molecular in AS progression. SETDB2 is a histone lysine methyltransferase and catalyzes trimethylation of H3K9 (Torrano et al., 2019). Due to its function on inflammatory factors *Ccl2* and *Cxcl1*, SETDB2 is considered to play a regulatory role in monocyte and neutrophil recruitment (Kroetz et al., 2015; Schliehe et al., 2015). Recently, Zhang et al. (2021) utilized scRNA-seq to profile CD45^+^ cells from atherosclerotic plaques of bone marrow-transplanted mice, and found that the proportions of a monocytes cluster and a neutrophil cluster in *Setdb2*-deficient leukocytes were increased compared with WT leukocytes. Meanwhile, in *Setdb2*-deficient leukocytes, the expression of proinflammatory factors, such as *Cebpb*, *S100a8*, *S100a9*, *Ccr1*, and *Trem1*, and genes related to the unfolded protein response, including *Clec4e*, *Clec4d*, and *Clec4n*, were elevated in the monocyte/mocrophage clusters. Genes upregulated in *Setdb2*-deficient samples were enriched in cell apoptosis and atherosclerosis signaling, whereas downregulated ones were associated with impaired regulation of anti-inflammatory response. These findings strongly supported the postulation of Zhang et al. (2021) that SETDB2 deficiency in hematopoietic cells enable exacerbate inflammation and aggravate atherosclerosis.

Recently, Örd et al. (2021) studied AS-relevant non-coding genetic variation by combining single-nucleus ATAC-seq with genome-wide association study (GWAS). They provided the first chromatin accessibility map of human AS lesions at single-cell resolution and classified cells into different types, including ECs, SMCs, and monocyte/macrophages. They discovered that ECs and SMCs possessed most CAD-associated genetic variants, and optimized the identification of potential causal single-nucleotide polymorphisms (SNPs) and the identification of the target genes for over 30 CAD loci. This work complements the view previously presented by scRNA-seq study (Wirka et al., 2019), and provides a new approach to discover disease-leading or disease-relevant cell types and gene variants.

### Other Vascular Diseases

While atherosclerosis remains the major vascular disease that has been studied with single-cell technologies (Cochain et al., 2018; Winkels et al., 2018; Lin et al., 2019; Pan et al., 2020), multiple other vascular diseases have also received attention (Pedroza et al., 2020; Zhang et al., 2020). Chen et al. (2020) recently investigated the pathogenesis of aortic aneurysm using mass cytometry, imaging mass spectroscopy (IMC) and scRNA-seq. Aortic aneurysm is characterized by loss of elastin fibers, medial degeneration, and low-grade aortic wall inflammation (Isselbacher, 2005; Guo et al., 2006). In aortic aneurysms caused by genetic anomalies, such as Marfan and Loeys-Dietz syndromes, activation of TGF-β signaling in SMCs is the well-known molecular mechanism (Lindsay and Dietz, 2014). However, for older patients suffering from chronic vascular diseases, such as atherosclerosis and hypertension, the pathogenesis of aortic aneurysm may be more complicated. With the combined use of multiple single-cell techniques, Chen et al. (2020) demonstrated the existence of a distinct SMC-derived mesenchymal stem cell (MSC)-like cell population caused by ablation of TGF-β signaling, which then differentiated into several mesenchymal lineage cell types, including adipocytes, chondrocytes, osteoblasts, and macrophages. These transformations led to the degradation of ECM, cartilage and bone formation, extensive lipid storage, and serious inflammation that ultimately gave rise to aortic aneurysm. This work demonstrated the advantage of combinational use of single-cell methods in mapping cell fate conversions responsible for disease onset.

Hypertension is a top risk factor for many cardiovascular diseases (Doyle, 1991). Vascular remodeling results in increased vascular resistance, which is a central event in hypertension, but its molecular underpinnings are still unclear. Previous studies primarily focused on EC dysfunction and phenotypic switching of SMCs (Brown et al., 2018; Touyz et al., 2018; Barman et al., 2019; Helmstädter et al., 2020). Using scRNA-seq, Cheng et al. systematically depicted artery type-specific gene expression changes in all major cell types (e.g., SMC, EC, MSC) and compared cell-cell communication changes in spontaneously hypertensive rats, which was not detected with previous conventional approaches (Cheng et al., 2021). This included discovering interaction of *Eng* with various growth factors of TGF signaling pathway, such as *Tgfb1*, *Tgfb3*, and *Bmp2*, in hypertension rats. Eng is a component of the TGF-β superfamily of receptors. The Eng-mediated crosstalk of TGF-β pathway regulates the function of vascular endothelial cells and angiogenesis *in vivo* (Tian et al., 2012). Variations of the *Eng* was found in pulmonary arterial hypertension patients, indicating the important role of *Eng* in hypertension (Uznañska-Loch et al., 2018).

## Clinical Value of Single-Cell Omics

Single-cell technologies provide an unprecedented opportunity to systematically uncover the cellular heterogeneity and dynamic molecular events during tissue development and disease progression. Clinical medicine for cardiovascular diseases may benefit from such technological advancements in many aspects.

Firstly, the study of dynamic cellular changes during disease onset and progression may yield promising candidates for biomarkers of diagnosis. At present, many diseases, such as the aortic dissection and aortic aneurysm, are still in need of specific and sensitive diagnostic tests. Single-cell sequencing is a good way to select and identify new biomarkers for acute aortic dissection, which may help doctors make timely decisions in the clinic (Suzuki et al., 2010; Suzuki and Eagle, 2018).

Flow cytometry, immunohistochemistry (IHC) and immunofluorescence (IF) are important tools for the clinical diagnosis of various diseases, particularly infectious diseases and, for the assessment of immune system function (Peters and Ansari, 2011; Yamanaka et al., 2018; Jain et al., 2019). However, these approaches are limited by the low number of parameters that can be analyzed simultaneously. Accordingly, high-dimensional approaches such as CyTOF and IMC may hold great potential for immune-monitoring and clinical diagnosis of cardiovascular diseases in the future. For example, CyTOF may prove useful in the subclassification of atherosclerosis or hypertension based on particular immune cell subtypes in peripheral blood, offering more accurate diagnosis and therapy.

Single-cell techniques are undeniably useful at identifying new therapeutic targets, which may be masked at conventional resolution. For instance, with CyTOF, Taverna J.A. et al. (2020) have proposed that combined inhibition of AXL and JAK1 may be a new therapeutic target for lung tumor. Currently, anti-hypertension therapies rely on suppressing the overactivated sympathetic nervous system and renin-angiotensin-aldosterone system. However, 10–30% hypertensive patients still remain insensitive even to the combined use of current anti-hypertensive medications (Cai and Calhoun, 2017). Under these circumstances, the use of single-cell sequencing may detect disease-specific cell subpopulations or cellular interactions that are crucial to disease pathogenesis, and thus may entirely circumvent therapy resistance. Alternatively, a comparative study of responsive versus non-responsive patients may help identify the molecular targets that confer resistance, with which one could devise an adjuvant therapy that increases treatment sensitivity.

## Conclusion

In the past few years, the field of single-cell biology has witnessed the rapid development of many single-cell omics techniques that are aimed to dissect all possible levels of cell biology. Their combinations have already yielded much insight into the spatial distribution and cellular heterogeneity of human fetal hearts at different stages, and the dynamic changes of gene expression patterns in cardiovascular diseases. Yet this comes with exponentially increasing challenges for data scientists. While Seurat v3 (Welch et al., 2019) and LIGER (Stuart et al., 2019) already perform reasonably well at integrating multiple data modalities, newer algorithms that better cope with single-cell multimodal data will be continuously sought after. Successful examples include Multi-Omics Factor Analysis v2 (MOFA+) (Argelaguet et al., 2020) and BIOMEX (Taverna F. et al., 2020), amongst others. Viewing the current trends in single-cell technology development, it seems possible that more advanced single-cell multi-omic techniques that capture spatial or *in situ* real-time information are next in line. Such techniques would be enormously useful at deciphering organ physiology and pathology. From a translational perspective, as single-cell techniques become increasingly widespread and ‘down-to-earth,’ one can envision them integrated into the diagnosis of certain cardiovascular diseases that are difficult to classify based on current knowledge. One such example could be dilated cardiomyopathy. Currently, dilated cardiomyopathy is one of the most complicated cardiac diseases. While clinical manifestations are relatively clear-cut, their etiologies, as well as cellular compositions and arrangements, can differ wildly from patient to patient. Application of single-cell genomics and transcriptomics may help trace down the genetic origin of the disease, while spatially resolved protein expression may aid in the classification of disease phenotype to facilitate implementation of precision medicine. With these tools at hand, single-cell approaches are expected to renew our knowledge of cardiovascular biology and diseases, and advance precision medicine and decision-making in the clinic in the foreseeable future.

## Author Contributions

YC, YL, and XG were all contributors to various parts of the review and helped in the writing of this review. All the authors contributed to the article and approved the submitted version.

## Conflict of Interest

The authors declare that the research was conducted in the absence of any commercial or financial relationships that could be construed as a potential conflict of interest.

## Publisher’s Note

All claims expressed in this article are solely those of the authors and do not necessarily represent those of their affiliated organizations, or those of the publisher, the editors and the reviewers. Any product that may be evaluated in this article, or claim that may be made by its manufacturer, is not guaranteed or endorsed by the publisher.
